# Associations between Progression of Retinal Pigment Epithelial and Outer Retinal Atrophy and Choroidal Thickness: A 2-Year observation

**DOI:** 10.1016/j.xops.2025.100939

**Published:** 2025-09-15

**Authors:** Norihiro Nagai, Hajime Shinoda, Hisashi Matsubara, Hiroto Terasaki, Takao Hirano, Aki Kato, Akiko Miki, Hiromasa Hirai, Fumiko Murao, Hiroko Imaizumi, Fumi Gomi, Yoshinori Mitamura, Nahoko Ogata, Sentaro Kusuhara, Tsutomu Yasukawa, Toshinori Murata, Taiji Sakamoto, Mineo Kondo, Yoko Ozawa

**Affiliations:** 1 Department of Ophthalmology, Keio University School of Medicine, Tokyo, Japan; 2 Department of Ophthalmology, Mie University Graduate School of Medicine, Tsu, Japan; 3 Department of Ophthalmology, Kagoshima University Graduate School of Medical and Dental Science, Kagoshima, Japan; 4 Department of Ophthalmology, Shinshu University School of Medicine, Matsumoto, Nagano, Japan; 5 Department of Ophthalmology and Visual Science, Nagoya City University Graduate School of Medical Sciences, Nagoya, Japan; 6 Division of Ophthalmology, Department of Surgery, Kobe University Graduate School of Medicine, Kobe, Japan; 7 Department of Ophthalmology, Nara Medical University, Nara, Japan; 8 Department of Ophthalmology, Tokushima University Graduate School, Tokushima, Japan; 9 Department of Ophthalmology, Sapporo City General Hospital, Sapporo, Japan; 10 Department of Ophthalmology, Hyogo Medical University, Nishinomiya, Japan; 11 Department of Clinical Regenerative Medicine, Fujita Medical Innovation Center Tokyo, Tokyo, Japan; 12 Eye Center, Fujita Health University Haneda Clinic, Tokyo, Japan

**Keywords:** Age-related macular degeneration, Choroidal thickness, Geographic atrophy, Photoreceptor, Retinal pigment epithelium.

## Abstract

**Purpose:**

To evaluate the clinical course of retinal pigment epithelial and outer retinal atrophy (RORA) with best-corrected visual acuity (BCVA) and risk factors for rapid progression to explore the pathogenesis.

**Design:**

Retrospective observational study.

**Subjects:**

Data on eyes with fovea-involved RORA associated with age-related macular degeneration were collected over time from 10 hospitals in Japan.

**Methods:**

Data on ophthalmic examination, BCVA, and OCT images were analyzed.

**Main Outcome Measures:**

Relationships between changes in BCVA and extents of RORA and outer plexiform layer (OPL) deterioration and their associations with central choroidal thickness (CCT) and pachychoroid characteristics at baseline were evaluated.

**Results:**

Of the 53 eyes of 53 patients (mean age; 74.9 ± 1.4 years), 32 eyes (60.4%) belonged to men. The progression in the mean extent of OPL deterioration was evident at year 1, whereas that of RORA, BCVA impairment, thinning of the central retinal thickness, and CCT became apparent at year 2 (*P* < 0.05). Changes in the extents of RORA and OPL deterioration and BCVA were correlated (*P* < 0.05). Baseline CCT negatively correlated with baseline RORA and the changes in extent of RORA (*P* < 0.05). After adjusting for age and sex, a longer extent of RORA at baseline predicted BCVA worsening ≥0.04 per year (odds ratio [OR], 3.444; 95% confidence interval [CI], 1.015–11.691; *P* = 0.047). Greater horizontal extension of RORA ≥175 μm/y was frequently observed in eyes with thinner CCT <180 μm (OR, 4.684; 95% CI, 1.288–17.036; *P* = 0.019), subretinal drusenoid deposits (SDDs) (OR, 6.714; 95% CI, 1.555–28.988; *P* = 0.011), and drusen (OR, 4.392; 95% CI, 1.176–16.410; *P* = 0.028) and less observed in eyes with pachychoroid characteristics (OR, 0.038; 95% CI, 0.003–0.454, *P* = 0.010) at baseline after adjusting for age and baseline extent of RORA; similar risks for greater vertical extension of RORA were observed.

**Conclusions:**

The change in BCVA paralleled the changes in the extents of RORA and OPL deterioration. Rapid BCVA impairment was observed in eyes with longer RORA at baseline. A thinner choroid, SDD, and drusen were risk factors, and pachychoroid characteristics were protective factors against RORA progression. Further studies are warranted to better understand the progression of RORA and vision loss.

**Financial Disclosure(s):**

Proprietary or commercial disclosure may be found in the Footnotes and Disclosures at the end of this article.

Geographic atrophy (GA) is an unresolved cause of blindness worldwide. Despite the regulatory approval of anticomplement factor drugs for GA in the United States,^1^^,^^2^ the treatment is not widely employed. Moreover, owing to the insufficient understanding of its clinical course and pathogenesis, the management of GA has not been standardized. OCT is an imaging technology that is widely utilized for the clinical evaluation of the macula. In OCT images, choroidal hypertransmission becomes increasingly apparent with the progressive attenuation of photoreceptor (PR) and retinal pigment epithelial (RPE) cells. Therefore, the Classification of Atrophy Meeting Group developed the RPE and outer retinal atrophy (RORA) criteria for defining atrophy on the basis of OCT findings.^3^^,^^4^

In eyes with fovea-involved RORA, the best-corrected visual acuity (BCVA) varies, with some eyes having relatively good BCVA; eyes with greater extent of RORA exhibited worse BCVA impairment.^5^ However, the previous study was a cross-sectional study;^5^ thus, longitudinal observations of individual eyes are necessary to confirm the structure–function correlations.

The concepts of a thick choroid and a pachychoroid have increasingly received interest, particularly in relation to neovascular age-related macular degeneration.^6^, ^7^, ^8^, ^9^ Pachychoroid constitutes a negative factor for the resolution of exudative changes after 3 induction treatments with anti-VEGF drugs.^10^ However, the visual outcome does not necessarily differ between pachychoroid-related and nonpachychoroid-related polypoidal choroidal vasculopathy.^11^ Moreover, the genetic characteristics of pachychoroid neovasculopathy may indicate intermediate features between that of age-related macular degeneration and healthy eyes.^7^ Recent observational studies have revealed that GA occurs in eyes with pachychoroid characteristics;^12^, ^13^, ^14^ clinicians may encounter patients with GA with or without pachychoroid characteristics in daily practice.

This study aimed to evaluate OCT image–based changes that show the progression of atrophic lesions and changes in BCVA in eyes with GA with or without pachychoroid characteristics over ≥2 years. The associations between these changes and choroidal conditions were evaluated. The results will help understand the pathogenesis and estimate the prognosis of the patients with GA.

## Methods

This study adhered to the tenets of the Declaration of Helsinki and was approved by the Ethics Committee of Keio University School of Medicine (approval number: 20221111). The need for written informed consent was waived, and an opt-out approach was used.

### Participants

This multicenter retrospective study included Japanese patients with RORA from 10 hospitals in Japan (Keio University Hospital, Mie University Hospital, Kagoshima University Hospital, Shinshu University Hospital, Nagoya City University Hospital, Kobe University Hospital, Nara Medical University Hospital, Tokushima University Hospital, Sapporo City General Hospital, and the Hospital of Hyogo College of Medicine). Based on the Classification of Atrophy Meeting Group–proposed criteria, RORA was defined as eyes with (1) a region of signal hypertransmission into the choroid; (2) a corresponding zone of RPE attenuation or disruption; and (3) evidence of overlying PR degeneration in OCT images, without scrolled RPE or other signs of an RPE tear; if the extent of hypertransmission with degeneration of the RPE and PRs is >250 μm, it is defined as complete RORA (cRORA), and if these OCT signs were present but did not fulfill all the criteria for cRORA, it is defined as incomplete RORA^3^^,^^4^ The eyes which showed cRORA or incomplete RORA at baseline were included in the study. We refer to the Guideline for *Diagnostic Criteria for Atrophic Age-related Macular Degeneration* published by the Japanese Opthalmological Society,^15^ and eyes with the OCT findings of clear delineation and hypopigmentation or RPE depigmentation, together with clearly visible choroidal large vessels,^15^^,^^16^ were included in this study. Patients with hereditary diseases, retinal vascular occlusion, or fibrovascular pigment epithelial detachment were excluded, as were those with missing data. In patients whose both eyes were eligible, the eye with the greatest extent of RORA on the horizontal OCT section was included in the study.

### Eye Examinations

The patients underwent ophthalmic examinations, including BCVA, fundus photography, spectral-domain OCT, and fundus autofluorescence imaging.

### OCT

OCT images obtained using a spectral-domain OCT system (Spectralis OCT, Heidelberg Engineering GmbH; Optovue: Visionix; or Cirrus, Carl Zeiss Meditec, Inc) at each institute were analyzed as previously reported.^5^ Briefly, measurement methods in the OCT images passing through the fovea were obtained using a built-in protocol of a line 30° scan (Spectralis OCT), 5 mm × 5 mm retinal map scan (Optovue), and line 6-mm scan (Cirrus).^5^ The extent of RORA corresponded with hypertransmission, whereas outer plexiform layer (OPL) deterioration was determined by decreased intensity, discontinuity, and thinning of OPL findings within a 6000-μm diameter from the fovea. The extents of RORA and OPL deterioration were measured in the horizontal and vertical sections, and central retinal thickness (CRT) and central choroidal thickness (CCT) were in horizontal sections all passing through the fovea using a built-in caliper and the proprietary software that was provided with the device (N.N. measured and Y.O. confirmed the data). Presence of drusen and subretinal drusenoid deposits (SDDs) were determined by fundus photographs and OCT images. OCT images of horizontal and vertical sections were obtained from 53 and 50 eyes, respectively.

### Statistical Analyses

IBM SPSS Statistics (version 29.0; IBM Corp) was used for all the statistical analyses. Generalized mixed-model analyses, Pearson correlation coefficient analysis, univariate analyses, multiple logistic regression, and the Mann–Whitney *U* test were used to analyze the data. Statistical significance was set at *P* < 0.05. All data are presented as mean ± standard error values.

## Results

Of the 53 eyes of 53 patients who had fovea-involved RORA and were followed up for ≥2 years, 32 eyes (60.4%) belonged to men. At baseline, the mean age of the patients was 74.9 ± 1.4 (median, 77) years (Table 1); BCVA was 0.513 ± 0.064 (median, 0.398); extents of RORA in horizontal and vertical OCT sections were 2546 ± 288 and 2346 ± 250 (median, 2252 and 2078) μm, respectively; extents of OPL deterioration in horizontal and vertical sections were 1511 ± 199 and 1446 ± 206 (median, 1271 and 1038) μm, respectively; CRT was 130 ± 8 (median, 129) μm; and CCT was 202 ± 16 (median, 179) μm.

**Table 1:** Characteristics of the Eyes

Parameters	Value
n	53
Age	32 (60.4)
Male	74.9 ± 1.4 (45–89, 77)
BCVA (LogMAR)	0.513 ± 0.064 (–0.079 to 2.000, 0.398)
Extent of RORA (μm)	
Horizontal section	2546 ± 288 (110–11789, 2252)
Vertical section∗	2346 ± 250 (99–9381, 2078)
Extent of OPL deterioration (μm)	
Horizontal section	1511 ± 199 (0–7180, 1271)
Vertical section∗	1446 ± 206 (0–8876, 1038)
Central retinal thickness (μm)	130 ± 8 (10–233, 129)
Central choroidal thickness (μm)	202 ± 16 (17–621, 179)
Subretinal drusenoid deposits	14 (26.4)
Drusen	28 (52.8)

BCVA = best-corrected visual acuity; LogMAR = logarithm of the minimum angle of resolution; OPL = outer plexiform layer; RORA = retinal pigment epithelial and outer retinal atrophy.

Data are presented as mean ± standard error (range, median).

∗ n = 50.

**Figure 1:**
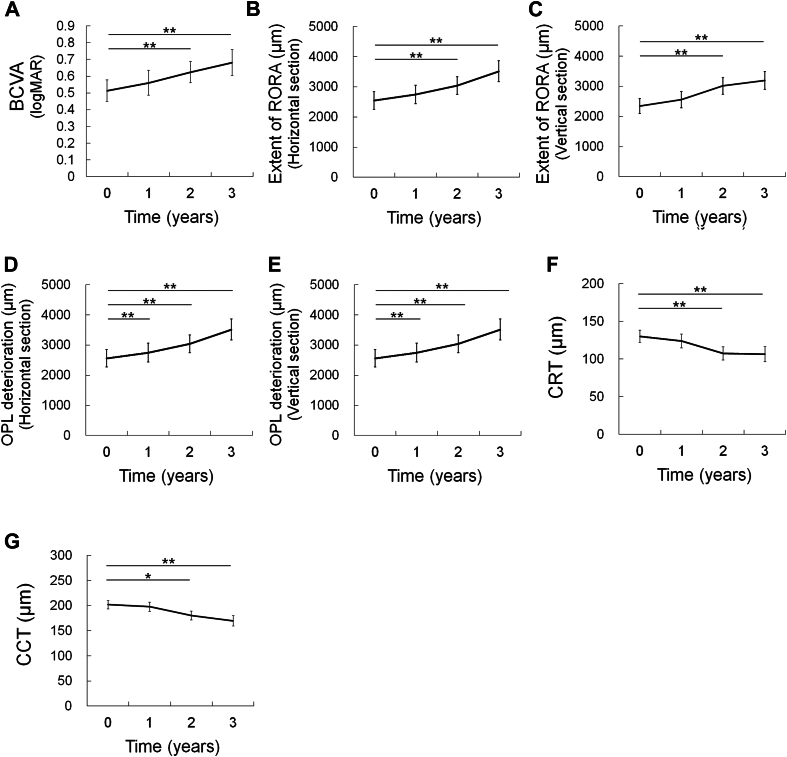
Changes in parameters over time. Plot of the mean values over time. (**A**) Best-corrected visual acuity; (**B**, **C**) extent of RORA; and (**D**, **E**) extent of OPL deterioration in horizontal and vertical sectional OCT images. (**F**) Central retinal thickness and (**G**) CCT. Generalized mixed-model analyses. ∗*P* < 0.05, ∗∗*P* < 0.01. BCVA = best-corrected visual acuity; CCT = central choroidal thickness; CRT = central retinal thickness; LogMAR = logarithm of the minimum angle of resolution; OPL = outer plexiform layer; RORA = retinal pigment epithelial and outer retinal atrophy.

Figure 1 depicts the data at baseline and at 1, 2, and 3 years. Compared with baseline, the mean BCVA worsened at years 2 and 3 (*P* < 0.001; Fig 1A), the mean extent of RORA increased in the horizontal and vertical sections at 2 and 3 years (*P* < 0.001 for all; Fig 1B and C), the mean extent of OPL deterioration increased at year 1 (*P* = 0.003 for the horizontal and vertical sections) and at years 2 and 3 (*P* < 0.001 for all sections; Fig 1D and E), the mean CRT decreased at years 2 and 3 (*P* = 0.002 for both; Fig 1F), and the mean CCT decreased at years 2 and 3 (*P* = 0.016 and *P* < 0.001, respectively; Fig 1G). Fifty-two eyes (98.1%) exhibited cRORA in which the extents of the OCT signs were >250 μm in horizontal or vertical sections, or in both, and 1 eye (1.9%) exhibited incomplete RORA in which the extents were <250 μm in both sections at baseline, while all eyes exhibited cRORA at 2 years.

Data recorded at 2 years were compared with those at baseline, and the changes per year were calculated; the results showed mean (median) increases of 246 (173) and 333 (177) μm in the respective horizontal and vertical extents of RORA, and an 11-μm mean reduction in CCT (Supplementary Table 1, available at www.ophthalmologyscience.org).

**Figure 2:**
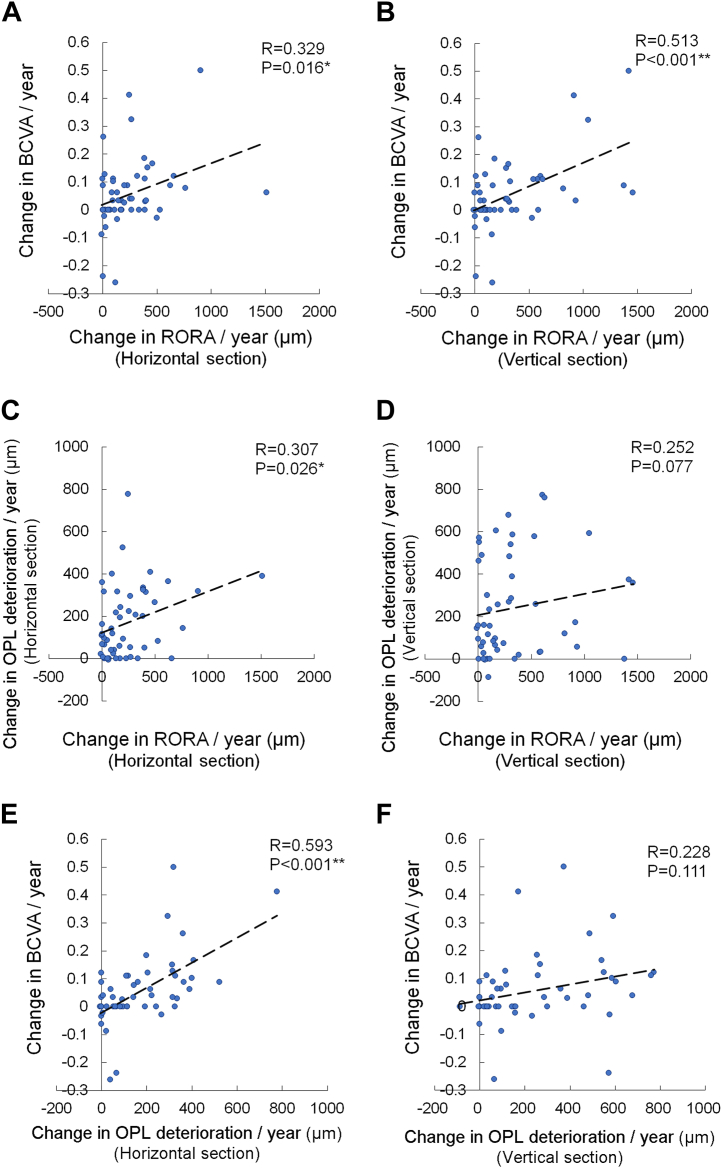
Correlations between changes in parameters per year. Correlations between changes in BCVA and extents of RORA in horizontal (**A**) and vertical (**B**) sections, changes in extent of RORA and OPL deterioration in respective horizontal (**C**) and vertical (**D**) sections, and changes in BCVA and OPL deterioration in respective horizontal (**E**) and vertical (**F**) sections. Pearson correlation coefficient analysis. ∗*P* < 0.05, ∗∗*P* < 0.01. BCVA = best-corrected visual acuity; OPL = outer plexiform layer; RORA = retinal pigment epithelial and outer retinal atrophy.

The changes in the horizontal (R = 0.329, *P* = 0.016; Fig 2A) and vertical (R = 0.513, *P* < 0.001; Fig 2B) extents of RORA per year from baseline correlated with the changes in the BCVA per year. Moreover, the change in the horizontal extent of RORA correlated with the change in the horizontal extent of OPL deterioration (R = 0.307, *P* = 0.026; Fig 2C), whereas the relationship showed a trend in the vertical section (R = 0.252, *P* = 0.077; Fig 2D). The change in the horizontal extent of OPL deterioration correlated with the change in the BCVA (R = 0.593, *P* < 0.001; Fig 2E), whereas the relationship in the vertical extent showed a trend (R = 0.228, *P* = 0.111; Fig 2F).

**Figure 3:**
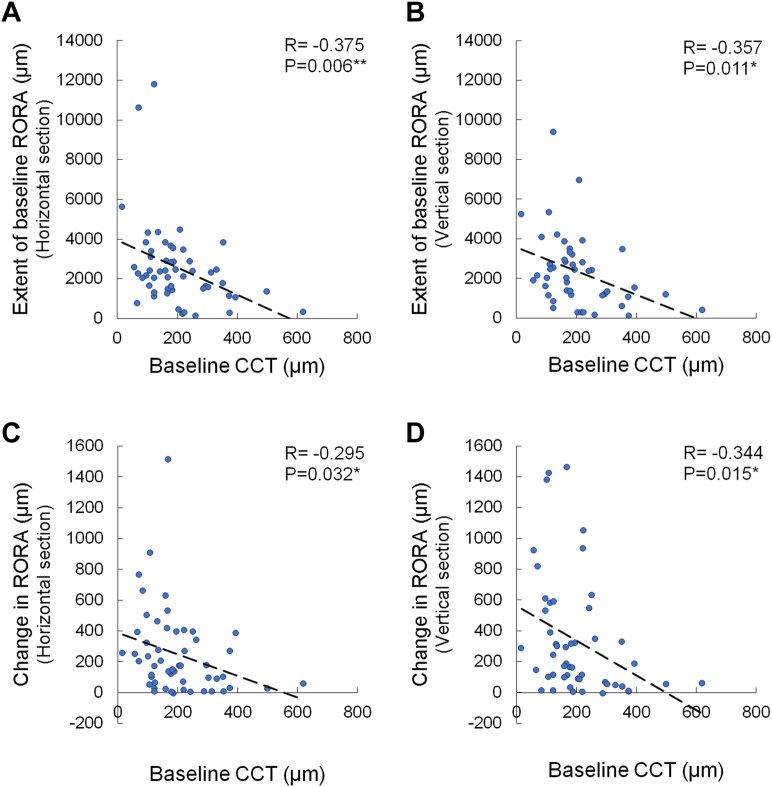
Correlations between baseline CCT and extents of RORA. Correlations between CCT and extent of RORA at baseline (**A**, horizontal section; **B**, vertical section) and between baseline CCT and changes in extent of RORA (**C**, horizontal section; **D**, vertical section). Pearson correlation coefficient analysis. ∗*P* < 0.05, ∗∗*P* < 0.01. CTT = central choroidal thickness; RORA = retinal pigment epithelial and outer retinal atrophy.

The CCT at baseline negatively correlated with the extent of RORA at baseline in both the horizontal and vertical sections (R = –0.375, *P* = 0.006 and R = –0.357, *P* = 0.011; Fig 3A and B, respectively) and with the change in the extent of RORA in both the horizontal and vertical sections (R = –0.295, *P* = 0.032 and R = –0.344, *P* = 0.015; Fig 3C and D, respectively).

Next, the factors associated with worsening of BCVA ≥0.04 in logarithm of the minimum angle of resolution per year until year 2 were compared with the baseline data (Table 2). The horizontal extent of RORA ≥2300 μm at baseline was a predictor of greater visual impairment after adjusting for age and sex (odds ratio [OR], 3.444; 95% confidence interval [CI], 1.015–11.691; *P* = 0.047). Factors associated with the extension of RORA ≥175 μm per year until year 2 were then analyzed. Progressions of greater horizontal and vertical extents of RORA were associated with CCT <180 μm at baseline after adjusting for age and extent of RORA at baseline (OR [95% CI] 4.684 [1.288–17.036], *P* = 0.019 and 3.982 [1.090–14.549], *P* = 0.037; Tables 3 and 4, respectively). Eyes with pachychoroid characteristics, which were represented by reduced fundus tessellation on color fundus photographs, dilated outer choroidal vessels on OCT, and no visible drusen, were observed in 12 eyes (22.6%) and comprised the negative risk of extending RORA ≥175 μm per year in the horizontal and vertical directions after adjusting for age and baseline extent of RORA (OR [95% CI] 0.038 [0.003–0.454], *P* = 0.010 and 0.114 [0.018–0.711], *P* = 0.020; Tables 3 and 4, respectively). The presence of SDD and drusen were associated with a greater extension of the RORA in the horizontal direction (SDD, OR [95% CI] 6.714 [1.555-28.988], *P* = 0.011; drusen, 4.392 [1.176-16.410], *P* = 0.028; Table 3) and vertical direction (SDD, OR [95% CI] 4.958 [1.054-23.332], *P* = 0.043; drusen, 4.270 [1. 161-15.697], *P* = 0.029; Table 4) after adjusting for age and baseline extent of RORA.

**Table 2:** Factors Associated with Change in BCVA ≥0.04 in LogMAR per Year

Characteristic	Crude			Age, Sex-Adjusted Logistic Regression Analyses		
	OR	95% CI	*P*	OR	95% CI	*P*
Male	0.322	0.103–1.012	0.052			
Age	1.015	0.962–1.072	0.585			
Extent of RORA ≥2300 μm in horizontal section	3.800	1.212–11.918	0.022∗	3.444	1.015–11.691	0.047∗
Extent of RORA ≥2100 μm in vertical section	3.187	0.999–10.171	0.050	2.602	0.775–8.738	0.122
BCVA ≥0.4	1.041	0.351–3.086	0.942			
CRT <140 μm	3.273	1.054–10.158	0.040∗	2.801	0.858–9.139	0.088
CCT <180 μm	2.361	0.779–7.155	0.129			
Pachychoroid GA	0.827	0.225–3.039	0.775			
SDD	1.917	0.557–6.596	0.302			
Drusen	1.103	0.373–3.261	0.859			

BCVA = best-corrected visual acuity; CCT = central choroidal thickness; CI = confidence interval; CRT = central retinal thickness; GA = geographic atrophy; LogMAR = logarithm of the minimum angle of resolution; OR = odds ratio; RORA = retinal pigment epithelial and outer retinal atrophy; SDD = subretinal drusenoid deposit.

∗ *P* < 0.05.

**Table 3:** Factors Associated with Extension of RORA ≥175 μm per Year in Horizontal Section

Characteristic	Crude			Age, Baseline RORA–Adjusted Logistic Regression Analyses		
	OR	95% CI	*P*	OR	95% CI	*P*
Male	0.513	0.168–1.566	0.241			
Age	1.005	0.953–1.060	0.865			
Extent of RORA ≥2300 μm in horizontal section	1.697	0.572–5.037	0.341			
BCVA ≥0.4	1.231	0.416–3.640	0.707			
CRT <130 μm	2.000	0.669–5.982	0.215			
CCT <180 μm	3.825	1.221–11.981	0.021	4.684	1.288–17.036	0.019∗
Pachychoroid GA	0.064	0.008–0.547	0.012	0.038	0.003–0.454	0.010∗
SDD	6.548	1.560–27.484	0.010	6.714	1.555–28.988	0.011∗
Drusen	3.284	1.059–10.186	0.040	4.392	1.176–16.410	0.028∗

BCVA = best-corrected visual acuity; CCT = central choroidal thickness; CI = confidence interval; CRT = central retinal thickness; GA = geographic atrophy; OR = odds ratio; RORA = retinal pigment epithelial and outer retinal atrophy; SDD = subretinal drusenoid deposit.

∗ *P* < 0.05.

**Table 4:** Factors Associated with Extension of RORA ≥175 μm per Year in Vertical Section

Characteristic	Crude			Age, Baseline RORA–Adjusted Logistic Regression Analyses		
	OR	95% CI	*P*	OR	95% CI	*P*
Male	0.844	0.269–2.647	0.771			
Age	1.017	0.957–1.081	0.591			
Extent of RORA ≥2100 μm in horizontal section	1.174	0.387–3.560	0.777			
BCVA ≥0.4	1.385	0.451–4.246	0.569			
CRT <130 μm	1.174	0.387–3.560	0.777			
CCT <180 μm	3.160	0.996–10.031	0.051	3.982	1.090–14.549	0.037∗
Pachychoroid GA	0.155	0.029–0.813	0.027	0.114	0.018–0.711	0.020∗
SDD	3.500	0.921–13.307	0.066	4.958	1.054–23.332	0.043∗
Drusen	3.857	1.180–12.606	0.025	4.270	1.161–15.697	0.029∗

BCVA = best-corrected visual acuity; CCT = central choroidal thickness; CI = confidence interval; CRT = central retinal thickness; GA = geographic atrophy; OR = odds ratio; RORA = retinal pigment epithelial and outer retinal atrophy; SDD = subretinal drusenoid deposit.

∗ *P* < 0.05.

In the presence of pachychoroid characteristics, the baseline RORA tended to be shorter in the horizontal section (*P* = 0.143) and was clearly shorter in the vertical section (*P* = 0.039) (Supplementary Table 2, available at www.ophthalmologyscience.org).

## Discussion

We demonstrated the changes in BCVA and extents of RORA and OPL deterioration over time in patients with RORA at baseline. Progression of OPL deterioration was apparent in year 1, whereas that of RORA and BCVA impairment was evident in year 2. The mean CRT and CCT were significantly reduced as early as the 2-year time point. Baseline CCT negatively correlated with the extents of baseline RORA and RORA progression. Data for changes per year showed that rapid worsening of BCVA was associated with a rapid extension of RORA and OPL deterioration; the changes in extents of RORA and OPL deterioration were consistently correlated. Baseline CCT negatively correlated with baseline and the change in the extent of RORA. Greater BCVA loss was predicted by a longer extent of baseline RORA; greater extension of RORA was frequently observed in eyes with thinner CCT and eyes with visible SDD or drusen at baseline, whereas lesser extension was noted in eyes with pachychoroid characteristics at baseline.

Overall, the progressive worsening of BCVA paralleled the increase in the extent of RORA and OPL deterioration as well as reduction in CRT. The extension of OPL deterioration may indicate the loss of PR synapses, and reduction in CRT may predominantly indicate the loss of PR cell body; the resulting PR damages allowed hypertransmission of the OCT signals, indicating the development and progression of RORA. The correlations between the progression of the functional and structural changes were clarified in the individuals over time.

**Figure 4:**
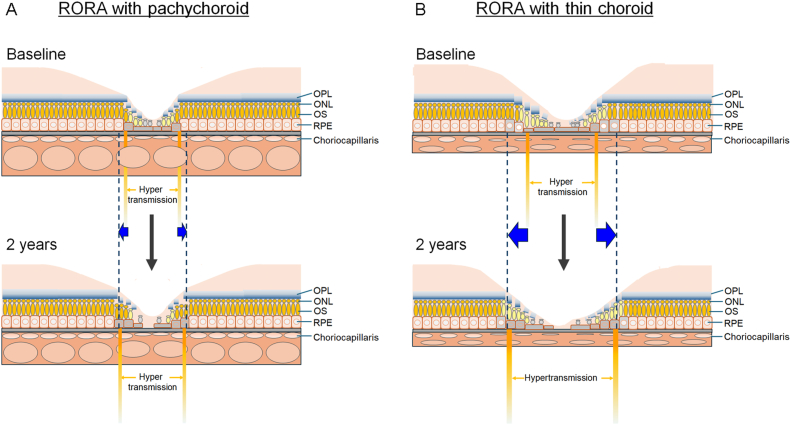
Hypothesis of the pathogenesis of RORA according to choroidal conditions. Findings at baseline and at 2 years were compared to hypothesize the progression process as follows. (**A**) In the eyes with pachychoroid, extent of RORA was relatively short at baseline. At this time point, RPE and photoreceptor cell loss may not be extremely severe, and some of the RPE cells may have enlarged to compensate the adjacent RPE cell loss. Microenvironment of the pachychoroid may slow the progressive vulnerability of the RPE and photoreceptor cells and extension of RORA. (**B**) In the eyes with thin choroid, extent of RORA at baseline was already relatively long. At this time point, many of the RPE cells had been lost and adjacent RPE cells may have enlarged and sustain a greater number of photoreceptor cells; the vulnerability may be advanced. As the RORA further progresses to efferent direction and extends, focal RPE and photoreceptor cell loss may be accelerated most likely due to advanced vulnerability, which leads to rapid progression. Note that blue arrows show the extension of RORA in 2 years. ONL = outer nuclear layer; OPL = outer plexiform layer; OS = photoreceptor outer segment; RORA = retinal pigment epithelial and outer retinal atrophy; RPE = retinal pigment epithelium.

The results of the current study showed that, after adjustment for age and sex, the worsening of BCVA over 2 years was related to a greater extent of RORA at baseline. This result aligns with that of a previous study where the ratio of lost lesions in PRs and RPE that were measured in OCT sectional images increased along with rapid GA progression.^17^ Given that BCVA persists for some time after foveal involvement in RORA,^5^ remaining PR cells, as observed in OCT images, may be functioning. This is most likely because the RPE cells can enlarge when adjacent cells are lost in order to compensate and maintain the RPE as an epithelial sheet and facilitate PR survival^18^ in the area of RORA (Fig 4A, B). Generally, a single RPE cell reportedly sustains 8 cone PRs.^19^ However, when RPE cells decrease, the adjacent and enlarged RPE cells may need to sustain a greater number of cone PR cells, which potentially confers additional stress on both cone PRs and RPE cells. Under these conditions, the function of each RPE cell, as well as that of the cone PRs, may become vulnerable and result in gradual retinal sensitivity impairment. Moreover, when RPE loss overwhelms the compensatory ability of the remaining RPE cells, a greater number of PR cells that were previously sustained by the remaining RPE cells may be lost at once, and this results in substantial loss of retinal sensitivity and BCVA (Fig 4B).

Interestingly, after adjusting for age and extent of RORA at baseline, the extension of RORA was related to a baseline thinner CCT (<180 μm). Eyes with larger areas of atrophy at baseline reportedly show larger enlargement rates.^20^^,^^21^ However, despite adjustment, the progression rate of the extent of RORA was more than fourfold greater in eyes with a thin choroid at baseline. Moreover, pachychoroid characteristics, which were not defined only by choroidal thickness, were a negative risk factor for RORA enlargement consistent with a previous report.^14^ Therefore, choroidal conditions may modify the risk of atrophic enlargement. A thick choroid is associated with hyperperfusion in eyes with hypertensive choroidopathy^22^ and central serous chorioretinopathy, ascertained using laser speckle-flow graphy.^23^ As choroidal flow delivers nutrients and oxygen to, and removes waste from, the RPE and PR cells, relatively persistent choroidal flow might slow the progressive vulnerability of the RPE and PRs in eyes with a thicker choroid or pachychoroid (Fig 4A).

Choroidal thickness can be affected by age,^24^ sex,^25^ and axial length.^26^, ^27^, ^28^ However, we compared the intraindividual CCT changes; sex did not change, and the axial length may not have changed substantially. In healthy eyes, the annual decrease in CCT is reportedly 3 μm,^24^ whereas the current study showed an 11-μm mean reduction in CCT per year, which suggests that eyes with RORA may experience rapid choroidal thinning. Given that VEGF secreted by the RPE is indispensable for the choroidal vasculature,^29^ progression of RORA and RPE degeneration may reduce local VEGF expression. Moreover, the decreased oxygen and nutrient demands of the atrophic RPE and outer retina may reduce choroidal flow and thickness.^30^^,^^31^ As the thin choroid itself may have further promoted atrophy, there would be a positive feedback cycle between the progression of RORA and choroidal thinning.

The presence of SDD and drusen were risk factors for the extension of RORA. These findings may be underestimated because they can be included in the area of the RORA and may not have been detected in fundus photographs. Nevertheless, these findings were related to a greater extension of the RORA, consistent with the previous clinical reports^32^^,^^33^ and the knowledge that they are distributed in eyes with PRs and RPE disorders,^19^ where their presence indicates tissue vulnerability. Moreover, the process of drusen-induced atrophy that was elucidated using OCT images obtained over time^34^ supports the results of this study.

The limitations of this study include the relatively small sample size, the retrospective nature of the study, and the fact that the OCT devices used were not the same for all eyes because the samples were collected from several institutes, and the differences of raster density among the OCT devices might cause differences in the measurements. However, the chronological changes were measured in the same institute and the measurement condition was sustained in individuals. Eyes with pachychoroid GA and the other GA were not separated in the main analyses. However, the correlations between baseline CCT and changes in extent of RORA was clear, suggesting that the clinicians may predict whether the patients will have rapid or slow progression by checking the CCT at baseline. The participants were all Japanese, and further studies to analyze whether the findings can be generalized in other populations would be required.

In conclusion, in eyes with fovea-involved RORA at baseline, BCVA worsened, the extent of RORA and OPL deterioration increased, and CRT and CCT decreased over time. The changes in BCVA and the extents of RORA and OPL deterioration were consistently correlated. Moreover, the relationship between the progression in the extent of RORA and baseline CCT was demonstrated. Rapid BCVA loss was observed in eyes with longer extent of RORA at baseline. Thinner choroid and the presence of SDD and drusen were risk factors for the rapid progression of RORA, and pachychoroid characteristics were relatively protective factors against RORA progression. The insights from this study facilitate the understanding of the clinical characteristics and pathogenesis of GA.

## Data Availability

The datasets generated during and/or analyzed during the current study are available from the corresponding author on reasonable request.
