# Supplementary material for: Associations between Progression of Retinal Pigment Epithelial and Outer Retinal Atrophy and Choroidal Thickness: A 2-Year observation
Source: Ophthalmol Sci. 2025 Sep 15;6(1):100939. doi: 10.1016/j.xops.2025.100939 (PMC12550781; doi:10.1016/j.xops.2025.100939)
Supplement: Supplementary Table 1 [file mmc1.pdf]

Supplementary Table 1. Changes in values per year

Parameters		Change/year
BCVA		0.056±0.017 (-0.261-0.500, 0.033)
Extent of RORA (μm)	Horizontal section	246±38 (-9-1513, 173)
	Vertical section†	333±54 (-10-1460, 177)
Extent of OPL deterioration (μm)	Horizontal section	170±22 (-6-778, 115)
	Vertical section†	239±32 (-88-772, 156)
CRT (μm)		-9±3 (-75-108, -10)
CCT (μm)		-11±2 (-59-27, -9)

Data are presented as mean ± standard error (range, median). †n=50. BCVA, best-corrected visual acuity; RORA, Retinal Pigment Epithelial and Outer Retinal Atrophy; OPL, outer plexiform layer; CRT, central retinal thickness, CCT, central choroidal thickness.
