# Supplementary material for: Associations between Progression of Retinal Pigment Epithelial and Outer Retinal Atrophy and Choroidal Thickness: A 2-Year observation
Source: Ophthalmol Sci. 2025 Sep 15;6(1):100939. doi: 10.1016/j.xops.2025.100939 (PMC12550781; doi:10.1016/j.xops.2025.100939)
Supplement: Supplementary Table 2 [file mmc2.pdf]

Supplementary Table 2. Comparisons between Pachychoroid GA and other GA

		Pachychoroid GA	Other GA	P
n (%)		12 (22.6)	41 (77.4)	
Male (%)		9 (75.0)	23 (56.1)	0.323
Age		69.2±4.2 (45-88, 73)	76.5±1.3 (57-89, 77)	0.101
BCVA	Baseline	0.398±0.136 (-0.079-1.222, 0.188)	0.547±0.073 (-0.079-2.000, 0.398)	0.237
	Change/year	0.047±0.025(-0.062-0.261, 0.017)	0.058±0.021 (-0.261-0.500, 0.033)	0.612
Extent of RORA (horizontal section)	Baseline (μm)	1733±309 (132-3819, 1690)	2784±358 (110-11789, 2383)	0.143
	Change/year (μm)	66±19 (1-176, 42)	299±46 (-9-1513, 248)	0.001**
Extent of RORA (vertical section)†	Baseline (μm)	1424±338 (99-3458, 1202)	2606±290 (151-9381, 2419)	0.039*
	Change/year (μm)	123±49 (5-543, 57)	392±64 (-10-1460, 287)	<0.001**
CRT	Baseline (μm)	134±14 (47-206, 133)	129±10 (10-233, 129)	0.758
	Change/year (μm)	5±10 (-27-108, -4)	-13±3 (-75-29, -12)	0.046*
Extent of OPL deterioration (horizontal section)	Baseline (μm)	922±261 (0-2318, 742)	1683±243 (0-7180, 1361)	0.093
	Change/year (μm)	127±41 (0-401, 103)	182±27 (-6-778, 142)	0.317
Extent of OPL deterioration (horizontal section)†	Baseline (μm)	1050±271 (0-2642, 1032)	1558±262 (0-8876, 1044)	0.444
	Change/year (μm)	138±53 (-5-587, 78)	268±39 (-88-772, 172)	0.085

Data are presented as mean ± standard error (range, median). †n=11 for Pachychoroid GA and n=39 for other GA. GA, geographic atrophy; BCVA, best-corrected visual acuity; RORA, Retinal Pigment Epithelial and Outer Retina Atrophy; CRT, central retinal thickness; OPL, outer plexiform layer. *P<0.05, **P<0.01.
